# Fault diagnosis model based on multi-strategy adaptive COA and improved weighted kernel ELM: A case study on wind turbine blade icing

**DOI:** 10.1371/journal.pone.0329332

**Published:** 2025-08-28

**Authors:** Xingtao Wu, Yunfei Ding, Ruizhi Zhao, Dong Ding, Hongwei Zhang, Lin Wang

**Affiliations:** 1 School of Electrical Engineering, Shanghai Dianji University, Shanghai, China; 2 State Grid Shanghai Changxing Power Supply Company, Shanghai, China; 3 Advanced Food Innovation Centre, Sheffield Hallam University, Sheffield, United Kingdom; 4 Aimsn Biomedical Technologies, Shanghai, China; Yalova University, TÜRKIYE

## Abstract

The icing failures of wind turbine blades are critical factors that affect both power generation efficiency and safety. To improve the diagnostic accuracy and speed, an improved weighted kernel extreme learning machine (IWKELM) optimized by multi-strategy adaptive coati optimization algorithm (MACOA) for icing fault diagnosis model is proposed, i.e., MACOA-IWKELM. Firstly, in order to improve the model optimization performance, the MACOA is proposed by introducing chaotic mapping Lévy flights, nonlinear inertial step factors, an improved coati vigilante mechanism, and an improved objective function. Secondly, the weighted kernel extreme learning machine (WKELM) is optimized by improved weighted parameters considering the influence of the internal distribution of samples on the diagnostic model. Finally, the MACOA is applied to the IWKELM and combined with the random forest (RF) dimensionality reduction technique to form the icing diagnostic model. The method is based on two sets of real SCADA data of wind turbine blade icing for comparison experiments with other models. In the two sets of experiments, the accuracy reaches 92.22% and 96.94% respectively, and the standard deviation of the accuracy in 50 experiments is 2.53% and 1.92% respectively. Keywords: Multi-strategy adaptive coati optimization algorithm; Improved weighted extreme learning machine; Wind turbine blade icing fault detection; Fault detection.

## Introduction

Wind energy is commonly used in various applications, including power generation, heating, and water pumping [1]. However, during the process of wind power generation, the turbine blades are susceptible to icing due to low-temperature conditions. Consequently, it is essential to study icing fault diagnosis. Currently, there are two primary categories of methods for diagnosing icing faults in wind turbine blades: mechanistic models and data-driven approaches [2].

Mechanistic models are based on physical and engineering principles to investigate the operational mechanisms and failure modes of wind turbines. However, these models tend to be complex, incur high computational costs, and pose challenges in terms of maintenance and updates. On the other hand, data-driven methods involve constructing intelligent models based on extensive datasets to detect and analyze the operational conditions of wind turbine blades, thereby assessing their operational status. This approach requires less specialized knowledge and has proven effective in actual predictive scenarios [3].

Common data-driven fault diagnosis methods are based on classifiers such as BP, ELM, KNN, SVM, and DT, among others [4]. While these methods have a well-established theoretical foundation and are cost-effective and widely applicable, they often depend on expert knowledge and face challenges in real-time monitoring, as well as the risk of misdiagnosis and omissions [5]. The Extreme Learning Machine (ELM) [6], proposed by Huang, is frequently employed in fault diagnosis due to its remarkable characteristics, including strong learning capability, effective testing performance, rapid training speed, and robust generalization ability. However, ELM exhibits limited generalization in nonlinear systems and is particularly sensitive to noise. To address these nonlinear issues, the Kernel-Based Extreme Learning Machine (KELM) was introduced [7]. Additionally, to tackle the problem of imbalanced data, Weighted Kernel-Based Extreme Learning Machine (WKELM) was proposed [8]. However, WKELM only applies weights to the two types of samples as a whole, overlooking the distribution within the samples, indicating that there is still room for improvement.

Since optimization algorithms can screen initial solutions for traditional models and improve their optimization search process, it is highly feasible and good diagnosis to use them to optimize fault diagnosis methods. Yan Y et al. [9] proposed an On-Load Tap-Changer fault diagnosis method based on the Weighted Extreme Learning Machine optimized by Improved Grey Wolf Algorithm. Guo X Y et al. [10] used an ELM model optimized by the Genetic Algorithm. In literature [11], the Grey Wolf Optimization-Ant Lion Optimizer-Extreme Learning Machine model was proposed. In the literature [12], a Kernel Extreme Learning Machine optimized by Grey Wolf Optimization was presented. The Coati Optimization Algorithm (COA) is a heuristic algorithm that simulates the natural behaviour of long-nosed coatis [13], has a strong optimization ability, which makes it competitive among similar algorithms. Jia et al. [14] proposed the introduction of a sound-based search encirclement strategy as well as a physical exertion strategy to improve the COA but failed to take into account the optimization of the generation of the initial population. Zhang et al. [15] improved the COA by applying it to real engineering problems, such as the three-bar truss design problem, but only a simple nonlinear strategy was used. Barak [16] proposed to combine the COA with the grey wolf optimization algorithm for active suspension linear quadratic regulator controller tuning. Baş [17] et al. proposed a nonlinear optimization algorithm ECOA (Enhanced Coati Optimization Algorithm). ECOA improved the COA by balancing exploitation capacity and exploration capacity but failed to consider eliminating the imbalance by optimizing the exploitation phase.

With the development of ELM, more and more models based on extreme learning machines have appeared. Tong R et al [18] proposed a new ellipsoid nearest neighbour graph computation strategy and fused ELM to form the ESS-ELM model. A short-term load forecasting model for distributed energy systems is introduced by the KELM optimized Fireworks Algorithm combining with Kernel Principal Component Analysis [19]. Vijaya et al [20] proposed a prediction model, which was combined with Variational Mode Decomposition and Multi Kernel Extreme Learning Machine Auto Encoder. Shang S et al [21] optimized the ELM by Improved Zebra Optimization Algorithm (IZOA). Pustokhina IV et al [22] used WELM model optimized by multi-objective rainfall optimization algorithm. Wang C L et al [23] proposed a sound quality prediction model based on ELM optimized by fuzzy adaptive Particle Swarm Optimization.

To address the issue of imbalanced wind turbine blade icing data, weighted parameters that vary according to the internal distribution of samples are introduced into the traditional Weighted Kernel Extreme Learning Machine (WKELM) model. This leads to the proposal of the Improved Weighted Kernel Extreme Learning Machine (IWKELM). In addition, to improve the performance of parameter optimization, this paper proposes a multi-strategy adaptive coati optimization algorithm (MACOA). The proposed MACOA uses a chaotic mapping mechanism to enhance the diversity and quality of the initial population. MACOA introduces a nonlinear inertial step size factor during the global optimization process to improve optimization efficiency. During the local optimization process, MACOA incorporates an improved sparrow vigilante mechanism to prevent the algorithm from falling into local optima. Additionally, an improved objective function is introduced during algorithm iteration to provide solutions for escaping local optima. Finally, MACOA is employed to optimize the parameters of the IWKELM model, resulting in the development of the MACOA-IWKELM icing diagnostic model. This model is compared with the BP, ELM, and KELM models, and experiments are conducted using the CEC2017 dataset, 12 publicly available datasets, and two sets of real turbine operation SCADA datasets to validate the effectiveness of the proposed method.

### Fundamental theories

*X*_*m×n*_ is an input data matrix which consists of *n* samples with *m* features. The *x*_*ij*_ denotes the jth feature value of the ith sample. The output matrix is defined as *Y*_*m×n*_.

### Weighted kernel extreme learning machine

According to the literature [6], the ELM is modelled as shown in Eq. (1) and Eq. (2):


$$Y=f(x)=\left\{ \begin{array}{c} h(x)H^{T}(I/C+HH^{T}{)}^{-1}T,whenn<L\\ h(x)(I/C+H^{T}H{)}^{-1}H^{T}T,whenn\geq L \end{array}\right.$$ (1)

$$H=[h(x_{1}{)}^{T},h(x_{2}{)}^{T},......,h(x_{n}{)}^{T}{]}^{T}={\left[\begin{array}{ccc} {}*20cg(w_{1}\cdot x_{1}+b_{1}) & \cdots & g(w_{L}\cdot x_{i}+b_{L})\\ \vdots & \ddots & \vdots \\ g(w_{1}\cdot x_{n}+b_{1}) & \cdots & g(w_{L}\cdot x_{n}+b_{L}) \end{array}\right]}_{n\times L}$$ (2)

where, the hidden layer output is defined as *h*(*x*_*i*_). the hidden layer matrix is *I*, whereas, *H* expresses the output matrix of the hidden layer neurons. *C* indicates the regularization parameter.

*T =* [*t*_*1*_, *t*_*2*_,...,*t*_*N*_]^T^ expresses the desired output of training sets. *L* represents the number of hidden layer neurons, and the internal parameters of the hidden neurons (*w*_*i*_ and *b*_*i*_) are randomly generated.

The kernel function *K*(*x*_*i*_, *x*_*j*_) is employed to solve the nonlinear mapping problem, shown in Eq. (3) [7]:


$$Y=f(x)=\left(\begin{array}{c} {}*20cK\left(x,x_{1}\right)\\ K\left(x,x_{2}\right)\\ \ldots \\ K\left(x,x_{N}\right) \end{array}\right)(I/C+\Omega {)}^{-1}T$$ (3)

$$K\left(x_{i},x_{j}\right)=\exp\left(\frac{-\left\|x_{i}-x_{j}\right\|}{g}\right)$$ (4)

where, the kernel matrix is *Ω* = *H*^*T*^*H*, *Ω*_*ij*_ expresses the element located in the ith row and jth column, and *K*(*x*_*i*_, *x*_*j*_) is the Gaussian kernel function as shown in Eq. (4).

When samples are trained using the traditional Kernel-Based Extreme Learning Machine (KELM), each sample is assigned equal importance. This approach significantly impacts the classification performance, particularly when there is interference from noise and outliers, or when the distribution of sample classes is highly imbalanced. To solve the problem, the WKELM model [8] is produced as shown in Eq. (5):


$$Y=f(x)=h(x)\beta =\left(\begin{array}{c} {}*20cK\left(x,x_{1}\right)\\ K\left(x,x_{2}\right)\\ \ldots \\ K\left(x,x_{N}\right) \end{array}\right){\left(I/C+W\Omega \right)}^{-1}WT$$ (5)

$$W=\left[\begin{array}{cccccc} {}*20cW_{+}(1) & & & & & \\ & \ddots & & & & \\ & & W_{+}(n_{1}) & & & \\ & & & W_{-}(1) & & \\ & & & & \ddots & \\ & & & & & W_{-}(n_{2}) \end{array}\right]$$ (6)

where *W* is the weighted matrix, the formula is shown in Eq. (6). *W*_*+*_(*i*) = *δ*_*1*_ and *W*_*-*_(*i*) = *δ*_*2*_ denote the weights of the positive and negative class samples, respectively.

### Coati optimization algorithm

The COA is a population intelligence optimization algorithm based on the behaviour of long-nosed coatis in nature [13]. In the COA, each individual coati is a candidate solution. They have two natural behaviours in the hunting period: (1) Hunting for iguana, (2) Escaping from predators. It can be interpreted in the algorithm as two phases: exploration and exploitation.

#### Hunting for iguana (exploration).

During the exploration phase, the coatis initiate a hunt and attack on the iguana, with a part of coatis climbing a tree in order to get close to the iguana. Other coatis wait beneath the tree to hunt the iguana once it fell to the ground. This strategy enables individual coatis to relocate to various positions within the search space, which demonstrates the global search capability of the COA within the problem space, i.e., Exploration.

During the exploration phase, $x_{best}^{t}$ denotes the position of the best individual in population, corresponds to the position of the iguana. Half of the coatis will ascend the tree, while the other half will remain on the ground, waiting for the iguana to fall. The position of the coati on the tree is shown in Eq. (7).


$$x_{i}^{t+1}(j)=x_{i}^{t}(j)+r\cdot \left(x_{best}^{t}(j)-RI\cdot x_{i}^{t}(j)\right),i=1,2,\cdot \cdot \cdot ,\frac{N}{2},j=1,2,\cdot \cdot \cdot ,M$$ (7)

where $x_{i}^{t}(j)$ is the position of an individual, *t* denotes the current iteration number, and *r* denotes a random number between [0,1]. *RI* denotes a random integer from {1,2}. *N* denotes the population size. *M* expresses the dimension.

After the iguana’s falling, it is placed randomly. Then, the coatis, which stay on the ground, move through the space, searching for the iguana. The position is updated by Eq. (8) and Eq. (9) below:


$$Iguana_{ground}^{t}(j)=lb_{j}+r\cdot (ub_{j}-lb_{j})$$ (8)

$$x_{i}^{t+1}(j)=\left\{ \begin{array}{c} x_{i}^{t}(j)+r\cdot \left(Iguana_{ground}^{t}(j)-I\cdot x_{i}^{t}(j)\right),iffitness(Iguana_{ground}^{t})<fitness(x_{i}^{t})\\ x_{i}^{t}(j)+r\cdot \left(x_{i}^{t}(j)-Iguana_{ground}^{t}(j)\right),else \end{array}\right.,i=\frac{N}{2}+1,\frac{N}{2}+2,\cdot \cdot \cdot ,N$$ (9)

where *lbj* and *ubj* expresses the lower and upper limit of the jth dimensional variable. *fitness*(·) is the formula for calculating fitness. $Iguana_{ground}^{t}$ expresses the new position of the iguana after falling. $x_{i}^{t}(j)$ is the value of the ith dimensional variable for the ith individual under the current iteration.

If the new position improves the fitness value, it is accepted as the new position. Otherwise, the coati remains in previous position, indicating that a greedy selection is performed shown in Eq. (10).


$$x_{i}^{t+1}=\left\{ \begin{array}{c} x_{i}^{t+1},iffitness(x_{i}^{t+1})<fitness(x_{i}^{t})\\ x_{i}^{t},else \end{array}\right.$$ (10)

#### Escaping from predators (exploitation).

During the exploitation phase, the updating of the coati’s location is modeled after the natural behavior of a coati escaping from a predator. This action allows the coati to move closer to a safer position nearby, reflecting the local search capability of the COA, which is indicative of exploitation.

During the exploitation phase, random positions are generated near every coati’s location, as shown in Eq. (11) and Eq. (12):


$$lb_{j}^{local}=\frac{lb_{j}}{t},ub_{j}^{local}=\frac{ub_{j}}{t},t=1,2,\cdot \cdot \cdot ,T$$ (11)

$$x_{i}^{t+1}(j)=x_{i}^{t}(j)-(1-2r)\cdot \left(lb_{j}^{local}+r\cdot (ub_{j}^{local}-lb_{j}^{local})\right),i=1,2,\cdot \cdot \cdot ,N$$ (12)

where *T* represents the maximum iteration count. *t* denotes the current number of iterations. $ub_{j}^{local}$ and $lb_{j}^{local}$ express the upper and lower bounds of the jth dimensional variable, which are updated with each iteration. *r* denotes a random number in the range of [0,1].

Finally, one more greedy choice is made, i.e., Eq. (10).

## Multi-strategy adaptive COA and improved weighted kernel ELM

### Multi-strategy adaptive coati optimization algorithm

#### Chaos mapping for Levi’s flight.

The chaotic mapping mechanism is characterized by high uncertainty and sensitivity. It can produce complex and unpredictable dynamic behaviors, allowing for a broader exploration of the search space [24,25]. Levy Flight is a specialized random walk model that describes movement patterns characterized by long-tailed distributions [26]. Levy flights are incorporated into the initialization process of the MACOA, as illustrated in Eqs. (13), (14) and (15):


$$\alpha ⊕Levi(\beta )\;\sim 0.01\frac{u}{{\left|v\right|}^{-\beta }}\left(\vec{X}(t)-\vec{X_{\alpha }}(t)\right)$$ (13)

$$\sigma_{u}={\left[\frac{\Gamma (1+\beta )\sin(\frac{\pi \beta }{2})}{\Gamma (\frac{1+\beta }{2})\beta \times 2^{\frac{\beta -1}{2}}}\right]}^{\frac{1}{\beta }},\sigma_{v}=1$$ (14)

X(t+1)=X(t)+α⊕Levi(β) (15)

where *X*(*t*) denotes the position of the ith coati, ⊕ expresses point-to-point multiplication, and *α* is the weight of the control step. $u\;\sim N(0,\sigma_{u}^{2})$. $v\;\sim N(0,\sigma_{v}^{2})$. *β* is the shape parameter of the step distribution, which is set to 1.5 in this paper.

#### Nonlinear inertia step size factor.

The introduction of a nonlinear inertia step size factor can significantly improve search efficiency and convergence performance, allowing the COA to dynamically adjust the search behavior. This mechanism maintains a high level of exploration capability during the initial stages, while the gradual reduction of weights in later stages encourages a more focused local search. Considering that updating a coati’s position is influenced by its current position, a nonlinear inertia step size factor is introduced. This factor adjusts the relationship between the coati’s position update and the current position information based on the individual coati’s location. The factor is then calculated using Eq. (16):


$$\omega =\frac{{\left(\frac{t}{T}\right)}^{Cn}}{{\left(\frac{t}{T}\right)}^{Cn}+{\left(1-\frac{t}{T}\right)}^{Cn}}$$ (16)

where *Cn* is a constant greater than 1 to control the degree of nonlinearities, which is taken as 2.

Initially, the value of *ω* is small, which means that position updates are less influenced by the current position. This allows for a broader search range for the algorithm and enhances its global exploration capability. As the search process progresses, the value of *ω* increases over time, resulting in a greater influence from the current coati position. This adjustment helps the algorithm in finding the optimal solution and also improves its convergence speed and local exploration ability.

The improved formula for modelling coati positions in the first stage is shown in Eq. (17):


$$x_{i}^{t+1}(j)=\omega \cdot x_{i}^{t}(j)+r\cdot \left(x_{best}^{t}(j)-I\cdot x_{i}^{t}(j)\right),i=1,2,\cdot \cdot \cdot ,\frac{N}{2}$$ (17)

#### Improved sparrow vigilante mechanism.

The Sparrow Search Algorithm is inspired by the behavior of sparrows while foraging for food, where some individuals act as vigilantes, responsible for monitoring their surroundings and sounding an alarm when a potential threat is detected. This approach enables the COA to maintain a higher degree of flexibility and dynamism in exploring the solution space, thereby enhancing its ability to adapt to uncertain problems [27].

Introducing the sparrow vigilante mechanism during the exploitation phase enhances the vigilance capability of the COA to search within an optimal range. Coatis at the edge of the population will quickly move away to find a safe area when they sense danger. Meanwhile, the coatis located in the center will move randomly to get closer to others in the population. The formula for the Sparrow Vigilante Mechanism is presented in Eq. (18):


$$X_{i,j}^{t+1}=\left\{ \begin{array}{c} X_{best}^{t}+\beta \cdot \left|X_{i,j}^{t}-X_{best}^{t}\right|,if\;f_{i}>f_{g}\\ X_{i,j}^{t}+K\cdot \left(\frac{\left|X_{i,j}^{t}-X_{worst}^{t}\right|}{(f_{i}-f_{W})+\varepsilon }\right),if\;f_{i}=f_{g} \end{array}\right.$$ (18)

where $X_{best}^{t}$ represents the global optimal position in the current iteration, *β* represents the step control parameter. *β ~ N*(0,1). *K* is a random number with values between [−1,1]. *f*_*i*_ is the fitness value. *f*_*g*_ is the global greatest fitness value, and *f*_*w*_ is the worst one. *ε* is a very small constant.

In order to escape from predation, coatis in the middle stay close to each other.

The Eq. (18) can be optimized to attack the problem of the global search capability. A dynamically adjusted step factor [28] is introduced shown in Eq. (19):


$$X_{i,j}^{t+1}=\left\{ \begin{array}{c} X_{best}^{t}+\beta (t)\cdot \left|X_{i,j}^{t}-X_{best}^{t}\right|,if\;f_{i}>f_{g}\\ X_{i,j}^{t}+K(t)\cdot \left(\frac{\left|X_{i,j}^{t}-X_{worst}^{t}\right|}{(f_{i}-f_{W})+\varepsilon }\right),if\;f_{i}=f_{g} \end{array}\right.$$ (19)

$$\beta (t)=f_{g}-(f_{g}-f_{w})\cdot (\frac{T-t}{T}{)}^{1.5}$$ (20)

$$K(t)=(f_{g}-f_{w})\cdot e^{-20\cdot \tan{\left(\frac{t}{T}\right)}^{2}}\cdot (2\cdot rand-1)$$ (21)

where *β*(*t*) is a dynamically adjusted step factor as shown in Eq. (20). *K*(*t*) is a dynamically adjusted step factor as shown in Eq. (21). *rand*∈[0,1].

The introduction of dynamic step factors *β*(*t*) and *K*(*t*) allows the algorithm to adjust its search behavior dynamically. In the initial stages of the algorithm, the focus is on exploration, while the later phases emphasize exploitation. These optimizations enhance the adaptability and robustness of the COA, particularly in complex and high-dimensional problems, enabling it to find the global optimal solution more efficiently.

#### Improved objective function.

Traditional objective functions often exhibit sensitivity to initial values, a tendency to converge on local optimal solutions, and a lack of robustness. Therefore, an improved objective function is proposed. In general, the dataset is divided into three subsets: the training set, the validation set, and the test set. Alternatively, it can be divided into two subsets: the training set and the test set. When the dataset is split into a training set and a test set, the objective function used to optimize the model parameters is either the number of classification errors (*ERROR*) or the root mean square error (*RMSE*) of the test results. *ERROR* and *RMSE* are calculated as shown in Eqs. (22) and (23).


$$ERROR=\frac{FP+FN}{TP+TN+FP+FN}$$ (22)

$$RMSE=\frac{1}{N}\sqrt{\sum_{i=1}^{N}(Y_{i}-T_{i}{)}^{2}}$$ (23)

When *ERROR* is used as the objective function, the particle can be viewed as approaching a decreasing extreme value during the reduction of the *ERROR*. However, there may be instances where, after reaching a certain extreme value, the particle fails to find a more optimal direction, leading to convergence at a local extreme value.

When *RMSE* is used as the objective function, it is possible for the *RMSE* value to decrease while the *ERROR* value increases. Although the overall direction of optimization is correct, the iteration may reduce the *RMSE* for the overall samples, resulting in most test samples being classified correctly. However, some samples may be misclassified in the next iteration, causing their classification results to change from correct to incorrect.

Therefore, an improved objective function is proposed, i.e., Eq. (24):


ERROR+ERMSE (24)

where *ERMSE* is the value of the root mean square for the error sample.

#### Multi-strategy adaptive coati optimization algorithm.

**Fig 1:**
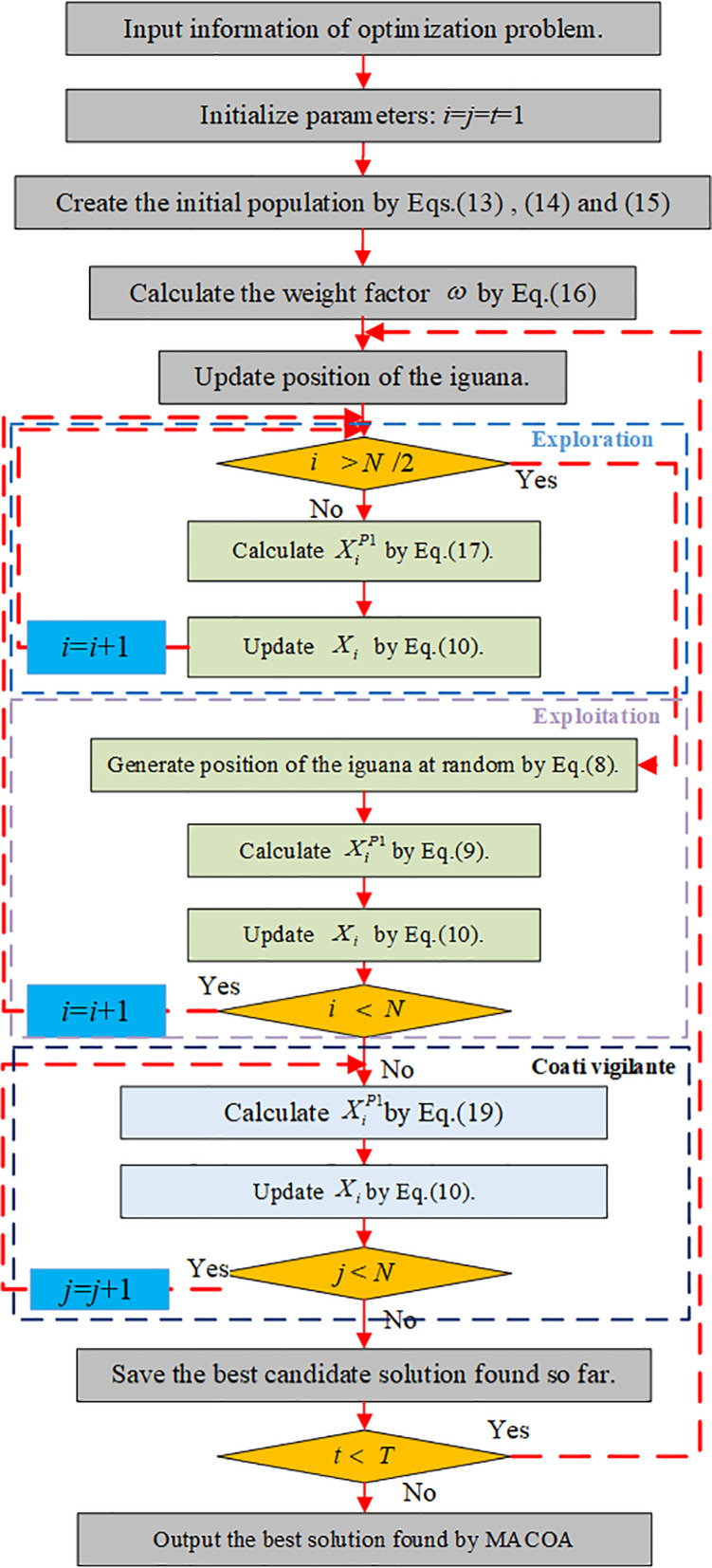
Flow chart of the MACOA.

The specific flowchart of the MACOA is shown in Fig 1.The pseudo-code for MACOA is shown in Table 1.

**Table 1:** Pseudo-code of MACOA.

Algorithm 1. Pseudo-code of MACOA.
**Start MACOA.** Input the optimization problem information. Set the number of iterations *T* and the number of coatis *N*. Initialization of all coatis and evaluation of the objective function for the population using Eqs. (13), (14) and (15). For t = 1:*T* Update location of the iguana based on the location of the best member of the population. **Phase 1:** Hunting and attacking strategy on the iguana (**Exploration Phase**) Calculate the weighted factor ω using Eq. (16) For i=1: [*N* / 2] Calculate new position for the ith coati using Eq. (17). Update position of the ith coati using Eq. (10). End for for i=N/2+1: N Calculate random position for the iguana using Eq. (8). Calculate new position for the ith coati using Eq. (9). Update position of the ith coati using Eq. (10). End for **Phase 2:** The process of escaping from predators (**Exploitation Phase**) For i = 1: N Calculate the new position for the ith coati using Eq. (19). Update the position of the ith coati using Eq. (10). End for Save the best candidate solution found so far End for **Output** of the best obtained solution by MACOA for given problem. **End MACOA.**

### Improved weighted kernel extreme learning machine

In the traditional Weighted Kernel Extreme Learning Machine (WKELM) model, the weighted parameter only influences the overall weight of each class of positive and negative samples. This approach results in the algorithm treating the two classes of samples as a whole during the optimization process, without considering the internal distribution of the samples. As a result, the information provided by the internal distribution is overlooked, which may negatively impact the model’s classification performance. To address this issue, the Improved Weighted Kernel Extreme Learning Machine (IWKELM) model is proposed. This model not only takes into account the overall weight distribution of the two types of samples but also focuses on the weights within each class, which vary according to their distribution, thereby enhancing the weighting of both types of samples.

For all positive sample weights, the formula was modified to Eq. (25):


$$W_{+}(i)=\left(d_{+}(i)/max(d_{+})*\delta_{1}+1\right)*\delta_{3}$$ (25)

For all negative sample weights, the formula was modified to Eq. (26):


$$W_{-}(i)=\left(d_{-}(i)/max(d_{-})*\delta_{2}+1\right)*(1-\delta_{3})$$ (26)

where *d*_*+*_(*i*) and *d*_*-*_(*i*) denote the Euclidean distance of the positive and negative samples to the centre of the respective two samples, and the formulae for the calculation of the respective centres of the two samples are given in Eq. (27) and Eq. (28).


$$d_{+}(i)=x_{center1}=\frac{1}{n_{1}}\sum_{i=1}^{n_{1}}x_{1i}$$ (27)

$$d_{-}(i)=x_{center2}=\frac{1}{n_{2}}\sum_{i=1}^{n_{2}}x_{2i}$$ (28)

For example, in the weighted formula for positive class samples, a term of (*d*_*+*_(*i*)/max(*d*_*+*_)*δ_*1*_+1) is introduced into the product, in addition to the weighted factor *δ*_*3*_, which affects all positive class samples. The term *d*_*+*_(*i*)/max(*d*_*+*_) is used to normalize the distances between the centers and all positive class samples. Meanwhile, the term (*d*_*+*_(*i*)/max(*d*_*+*_)*δ_*1*_+1) maps the normalized distances into the range of [1+*δ*_*1*_,1]. When multiplied by *δ*_*3*_, the distances between the centers and all positive class samples can be adjusted to [(1+*δ*_*1*_)*δ_*3*_,*δ*_*3*_].

Clearly, *δ*_*3*_ represents the upper limit of the weights for the positive class samples, while (1+*δ*_*1*_)*δ_*3*_ serves as the lower limit. *δ*_*3*_ is proportional to the total weights of the positive class samples and inversely proportional to the total weights of the negative class samples. Consequently, the closer a sample is to the center of the positive class, the closer its weight is to *δ*_*3*_. Conversely, as the distance increases, the weights of the edge-positive class samples approach (1+*δ*_*1*_)*δ_*3*_.

**Fig 2:**
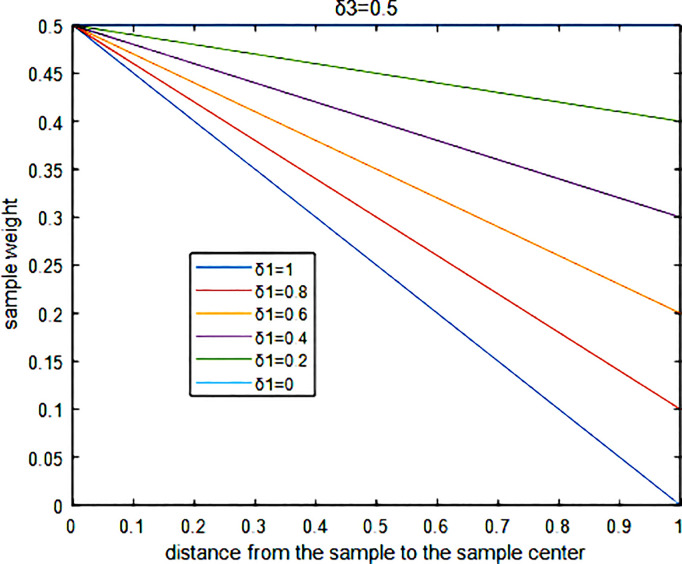
The relationship between the sample weight and the distance from the sample to the sample center.

For positive class samples, the relationship between the size of the sample weights and the distances from the samples to the sample centres is shown in Fig 2.

In Fig 2, after fixing *δ*_*3*_, it is evident that the closer the value of *δ*_*1*_ is to 0, the weights of all positive samples approach *δ*_*3*_, indicating that the internal distribution of the positive samples becomes less significant. Conversely, as the value of *δ*_*1*_ approaches −1, the weights of samples closer to the center of the positive class remain near *δ*_*3*_, while those further away from the center tend toward 0. This suggests that the influence of the internal distribution of positive samples still requires further consideration. Similarly, for negative samples, *δ*_*2*_ is related to the degree of influence exerted by the distribution of positions within the negative samples, and the overall weights of all negative samples are adjusted by controlling *δ*_*3*_.

To test the performance of IWKELM in handling the internal distribution of samples, marginal samples were taken from the KEEL dataset based on Z-score for experimentation. The specific experimental results are shown in Table 2.

**Table 2:** Experimental results for marginal sample sets.

	titanic_marginal	phoneme_marginal
BP	84.62%	71.77%
ELM	85.38%	72.54%
KELM	85.15%	79.15%
KNN	84.08%	77.46%
SVM	78.38%	66.85%
DT	85.62%	76.54%
COA-WKELM	86.08%	84.46%
MACOA-WKELM	86.69%	84.69%
COA-IWKELM	86.23%	85.15%
MACOA-IWKELM	86.77%	85.54%

The experimental results show that the diagnostic performance of the IWKELM model far exceeds that of traditional models. Furthermore, the diagnostic accuracy of COA-IWKELM is 0.15% and 0.69% higher than that of COA-WKELM in the two marginal data sets, respectively. The diagnostic accuracy of MACOA-IWKELM is 0.08% and 0.85% higher than that of MACOA-WKELM, respectively. The results show that IWKELM has a significant advantage in handling the internal distribution of samples.

**Fig 3:**
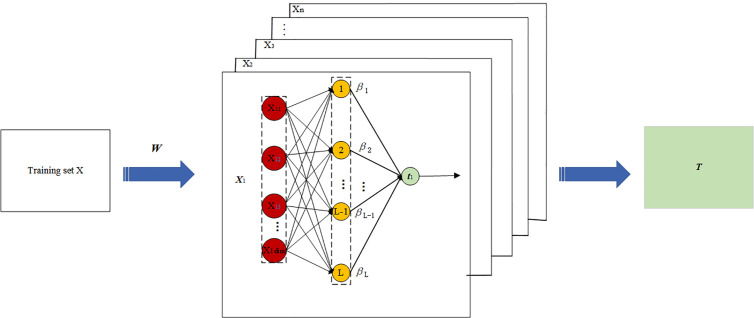
Structure chart of the IWKELM.

**Fig 4:**
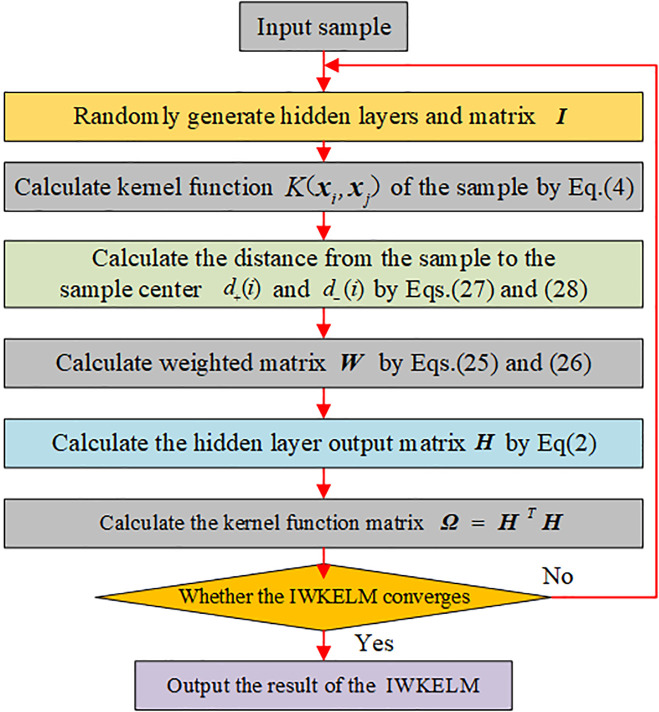
Flow chart of the IWKELM.

The specific structure of the modelling of the IWKELM is shown in Fig 3, and the flowchart is shown in Fig 4.

## Experiments for the multi-strategy adaptive coati optimization algorithm

This section presents simulation studies and evaluations of the optimization efficiency of the Multi-strategy Adaptive Coati Optimization Algorithm (MACOA). Given that the individual coatis in the proposed MACOA possess strong optimization capabilities, there is no need to set a large population for the algorithm. However, certain requirements exist regarding the number of iterations. Therefore, the experimental conditions, including the population size and the maximum number of iterations, are outlined in Table 3.

**Table 3:** Experiment condition.

Item	Parameter
CPU	AMD R7-5800H
RAM	16GB
Software	MATLAB R2018a
Population	20
Max iteration	1000

### Benchmark functions and compared algorithms

Twenty-nine standard benchmark functions from the IEEE CEC-2017 [29] have been utilized to evaluate MACOA’s capability in addressing various objective functions. A comparison of MACOA’s performance with eleven well-known algorithms is performed in order to assess its quality in providing optimal solutions, namely COA [13], SABO [30], WSO [31], SCSO [32], GJO [33], TSA [34], WOA [35], GWO [36], TLBO [37], GSA [38] and PSO [39]. The results are displayed using four metrics: mean, standard deviation (std), rank, and execution time (ET). The value of control parameters for all competing algorithms are detailed in Table 4.

**Table 4:** Values set for control parameters of compared algorithms.

Algorithm	Parameter	Value
COA	*r*: random number	*r*∈[0,1]
	*I*: random number	*I*∈[0,1]
SABO	*v*:random vector	*v*∈[1,2]
	*r*_*i*_: random number	*r*_*i*_ obeys a normal distribution
WSO	*f*_*min*_	0.07
	*f*_*max*_	0.75
	*τ*	4.11
	*a*_*0*_	6.25
	*a*_*1*_	100
	*a*_*2*_	0.0005
SCSO	*r*_*G*_	Linear reduction from 2 to 0.
	*S*_*M*_	2
GJO	*c*_*1*_	1.5
	*E*_*0*_: random number	*E*_*0*_∈[−1,1]
	*β*	1.5
TSA	*P*_*min*_	1
	*P*_*max*_	4
	*c*_*1*_,*c*_*2*_,*c*_*3*_	Random numbers stand in the interval[0,1]
WOA	*a*	Linear reduction from 2 to 0.
	*r*: random vector	*r*∈[0,1]
	*l*: random number	*l*∈[−1,1]
GWO	*a*	Linear reduction from 2 to 0.
TLBO	*T*_*F*_: teaching factor	*T*_*F*_ = round[(1 + rand)]
	*r*: random number	*r*∈[0,1]
GSA	Alpha	20
	*R*_*power*_	1
	*R*_*norm*_	2
	*G*_*0*_	100
PSO	Topology	Fully connected.
	*C*_*1*_: Cognitive constant	2
	*C*_*2*_: Social constant	2
	Inertia weight	Linear reduction from 0.9 to 0.1.
	Velocity limit	10% of the dimensions range of the variables.

### Experimental results and analysis

CEC-2017 includes thirty standard benchmark functions of various types, as shown in Table 5.

**Table 5:** Summary of the CEC-2017 test functions.

Name	No.	Functions	*Fi* = *Fi*(x*)
Unimodal Functions	1	Shifted and Rotated Bent Cigar Function	100
	3	Shifted and Rotated Zakharov Function	200
Simple Multimodal Functions	4	Shifted and Rotated Rosenbrock’s Function	300
	5	Shifted and Rotated Rastrigin’s Function	400
	6	Shifted and Rotated Expanded Scaffer’s F6 Function	500
	7	Shifted and Rotated Lunacek Bi_Rastrigin Function	600
	8	Shifted and Rotated Non-Continuous Rastrigin’s Function	700
	9	Shifted and Rotated Levy Function	800
	10	Shifted and Rotated Schwefel’s Function	900
Hybrid Functions	11	Hybrid Functions 1(N = 3)	1000
	12	Hybrid Functions 2(N = 3)	1100
	13	Hybrid Functions 3(N = 3)	1200
	14	Hybrid Functions 4(N = 4)	1300
	15	Hybrid Functions 5(N = 4)	1400
	16	Hybrid Functions 6(N = 4)	1500
	17	Hybrid Functions 6(N = 5)	1600
	18	Hybrid Functions 6(N = 5)	1700
	19	Hybrid Functions 6(N = 5)	1800
	20	Hybrid Functions 6(N = 6)	1900
Composition Functions	21	Composition Functions 1(N = 3)	2000
	22	Composition Functions 2(N = 3)	2100
	23	Composition Functions 3(N = 4)	2200
	24	Composition Functions 4(N = 4)	2300
	25	Composition Functions 5(N = 5)	2400
	26	Composition Functions 6(N = 5)	2500
	27	Composition Functions 7(N = 6)	2600
	28	Composition Functions 8(N = 6)	2700
	29	Composition Functions 9(N = 3)	2800
	30	Composition Functions 10(N = 3)	2900
Search Range:[−100,100]^D^			

The test function F2 from the CEC-2017 is not used in this paper because of its unstable performance (same as other authors in their paper [15]). Complete information and details for these test functions can be found in literature [29].

The proposed Multi-strategy Adaptive Coati Optimization Algorithm (MACOA) and baseline algorithms were subjected to 29 independent experiments at CEC-2017, each consisting of 200,000 function evaluations (FEs). The experiments utilized three dimensions of test functions: 30, 50, and 100. The ranking results for the experiments are presented in Tables 6–8. The results for the 30-dimensional case (m = 30) indicate that the MACOA is the best algorithm for solving the F4, F10, F11, F22, F24–F26, F28, and F29 functions.

**Table 6:** Rank results of the CEC-2017 objective functions (the dimension m = 30).

	MACOA	COA	SABO	WSO	SCSO	GJO	TSA	WOA	GWO	TLBO	GSA	PSO
F1	2	11	7	5	6	8	9	12	4	**1**	3	10
F3	2	10	4	7	5	8	6	12	3	**1**	11	9
F4	**1**	11	8	7	5	6	9	12	4	3	2	10
F5	2	11	7	3	6	4	9	12	**1**	8	5	10
F6	2	10	7	4	6	3	8	11	**1**	12	5	9
F7	2	11	7	6	5	4	9	12	**1**	8	3	10
F8	3	10	7	2	6	5	9	12	**1**	11	4	8
F9	4	10	7	9	6	5	11	12	**1**	2	3	8
F10	**1**	10	9	4	5	6	7	11	3	12	2	8
F11	**1**	11	8	3	5	7	9	12	4	2	6	10
F12	2	11	6	7	5	8	9	12	4	**1**	3	10
F13	3	12	6	5	7	8	10	11	4	**1**	2	9
F14	3	11	9	4	5	7	10	12	6	2	8	**1**
F15	4	11	7	**1**	8	9	10	12	6	2	3	5
F16	3	11	9	**1**	5	4	8	12	2	6	7	10
F17	5	12	9	**1**	4	2	8	11	3	6	7	10
F18	2	11	9	3	8	6	10	12	7	4	5	**1**
F19	3	11	7	2	8	9	10	12	5	**1**	4	6
F20	3	10	8	**1**	5	4	6	12	2	9	7	11
F21	2	9	6	4	5	3	8	11	**1**	12	7	10
F22	**1**	11	4	7	5	6	9	12	2	3	8	10
F23	2	8	6	5	4	3	7	10	**1**	9	11	12
F24	**1**	11	5	8	4	3	7	9	2	12	6	10
F25	**1**	11	7	5	4	6	8	12	3	9	2	10
F26	**1**	11	8	6	5	3	9	12	2	4	7	10
F27	3	11	7	8	6	5	9	2	4	10	12	**1**
F28	**1**	12	9	7	6	8	11	3	5	10	4	2
F29	**1**	11	9	3	5	4	6	12	2	8	7	10
F30	2	11	7	3	6	8	10	12	5	**1**	4	9
Sum rank	**63**	311	209	131	160	162	251	319	89	170	158	239
Mean rank	**2.172**	10.724	7.207	4.517	5.517	5.586	8.655	11	3.069	5.862	5.448	8.241
Total rank	**1**	11	8	3	5	6	10	12	2	7	4	9

**Table 7:** Rank results of the CEC-2017 objective functions (the dimension m = 50).

	MACOA	COA	SABO	WSO	SCSO	GJO	TSA	WOA	GWO	TLBO	GSA	PSO
F1	**1**	11	6	8	5	7	9	12	4	2	3	10
F3	6	11	9	4	**1**	3	2	12	5	8	10	7
F4	**1**	11	7	8	5	6	9	12	4	2	3	10
F5	2	11	8	4	6	5	10	12	**1**	7	3	9
F6	2	9	7	4	6	3	10	11	**1**	12	5	8
F7	2	11	6	7	5	3	9	12	**1**	8	4	10
F8	2	11	8	4	6	5	10	12	**1**	7	3	9
F9	3	10	8	9	4	6	11	12	2	5	**1**	7
F10	**1**	10	9	4	5	6	7	11	3	12	2	8
F11	**1**	11	5	3	6	7	8	12	4	2	9	10
F12	2	12	6	8	5	7	9	11	4	**1**	3	10
F13	2	12	6	8	5	7	9	11	4	**1**	3	10
F14	2	12	8	7	5	6	10	11	4	**1**	9	3
F15	2	11	5	7	6	8	10	12	4	**1**	3	9
F16	**1**	12	8	4	7	6	9	11	2	5	3	10
F17	2	11	8	3	6	4	9	12	**1**	7	5	10
F18	**1**	11	9	4	6	8	10	12	5	2	3	7
F19	3	12	7	4	5	8	10	11	6	**1**	2	9
F20	3	10	9	**1**	5	4	7	12	2	11	6	8
F21	2	10	7	4	5	3	8	12	**1**	11	6	9
F22	**1**	9	8	3	5	6	7	11	2	12	4	10
F23	**1**	10	6	5	4	3	7	9	2	8	11	12
F24	**1**	12	5	8	3	4	6	11	2	9	7	10
F25	**1**	11	8	6	5	7	9	12	4	2	3	10
F26	**1**	11	7	5	4	3	9	12	2	8	6	10
F27	3	11	7	8	6	5	9	**1**	4	10	12	2
F28	3	12	11	8	7	9	10	2	5	4	6	**1**
F29	**1**	11	9	3	6	4	8	12	2	5	7	10
F30	2	11	8	5	6	7	9	12	4	**1**	3	10
Sum rank	**55**	317	215	156	150	160	250	315	86	165	145	248
Mean rank	**1.897**	10.931	7.414	5.379	5.172	5.517	8.621	10.862	2.966	5.690	5	8.552
Total rank	**1**	12	8	5	4	6	10	11	2	7	3	9

**Table 8:** Rank results of the CEC-2017 objective functions (the dimension m = 100).

	MACOA	COA	SABO	WSO	SCSO	GJO	TSA	WOA	GWO	TLBO	GSA	PSO
F1	**1**	11	6	8	4	9	5	12	3	2	7	10
F3	5	6	4	3	**1**	7	10	12	9	11	8	2
F4	**1**	11	7	8	4	5	6	12	3	2	9	10
F5	2	10	8	4	6	5	11	12	**1**	7	3	9
F6	2	9	8	4	6	5	10	11	**1**	12	3	7
F7	2	11	6	8	5	3	9	12	**1**	7	4	10
F8	2	11	8	4	6	5	10	12	**1**	7	3	9
F9	2	9	7	8	3	5	11	12	4	10	**1**	6
F10	**1**	10	9	4	5	6	7	11	3	12	2	8
F11	4	11	9	6	2	7	3	12	5	**1**	8	10
F12	**1**	11	5	7	4	6	9	12	3	2	8	10
F13	2	11	6	8	4	7	9	12	3	**1**	5	10
F14	**1**	11	9	6	3	8	7	12	4	2	5	10
F15	2	11	5	8	6	7	9	12	3	**1**	4	10
F16	**1**	11	9	4	6	5	8	12	2	3	7	10
F17	**1**	11	6	7	4	5	9	12	3	2	8	10
F18	2	11	9	6	5	7	8	12	4	**1**	3	10
F19	2	11	6	7	4	8	9	12	3	**1**	5	10
F20	2	10	9	**1**	4	6	7	12	3	11	5	8
F21	2	10	9	5	4	3	6	11	**1**	7	8	12
F22	**1**	10	9	3	5	6	7	11	4	12	2	8
F23	**1**	11	7	5	4	3	8	9	2	6	12	10
F24	**1**	12	8	6	3	4	7	10	2	5	11	9
F25	**1**	11	7	9	4	8	6	12	3	2	5	10
F26	**1**	11	9	5	4	3	6	12	2	8	7	10
F27	3	12	8	10	5	6	9	2	4	7	11	**1**
F28	3	12	9	11	6	8	7	2	5	4	10	**1**
F29	**1**	11	7	5	4	6	8	12	3	2	9	10
F30	**1**	11	5	7	4	6	9	12	3	2	8	10
Sum rank	**51**	308	214	177	125	169	230	319	88	150	181	250
Mean rank	**1.759**	10.621	7.379	6.103	4.310	5.828	7.931	11	3.034	5.172	6.241	8.621
Total rank	**1**	11	8	6	3	5	9	12	2	4	7	10

The results for the 50-dimensional case (m = 50) clearly indicate that MACOA is the best optimization algorithm for solving the F1, F4, F10, F11, F16, F18, F22–F26, and F29 functions. Similarly, the results for the 100-dimensional case (m = 100) demonstrate that MACOA excels in solving the F1, F4, F10, F12, F14, F16, F17, F22–F26, F29, and F30 functions. A comparison of the experimental results shows that MACOA outperforms the competing algorithms for most of the tested functions. Overall, MACOA consistently delivers the best performance across different dimensions (30, 50, and 100) of the CEC-2017 test functions.

Compared with other 11 algorithms, the MACOA proposed has strong exploration, exploitation and search capability. It has superior performance compared to other optimization algorithms.

## Wind turbine blade icing fault diagnosis model based on MACOA-IWKELM

To enhance the diagnostic correctness of the IWKELM. A wind turbine blade icing diagnosis model based on MACOA-IWKELM is proposed. The specific process of modelling the model is as follows below:

(1) All wind turbine blade SCADA point data is adjusted and grouped out, overpowered samples are removed, some attributes are averaged, and then all data is normalized by the minimum-maximum standardization method.
(2) All data are processed using the Random Forest algorithm for dimensionality reduction to avoid too high dimensionality leading to too poor training results.
(3) The MACOA-IWKELM model is used for wind turbine blade icing fault diagnosis among the dataset obtained after the dimensionality reduction process, and a compared classification model is set up for experimentation.

**Fig 5:**
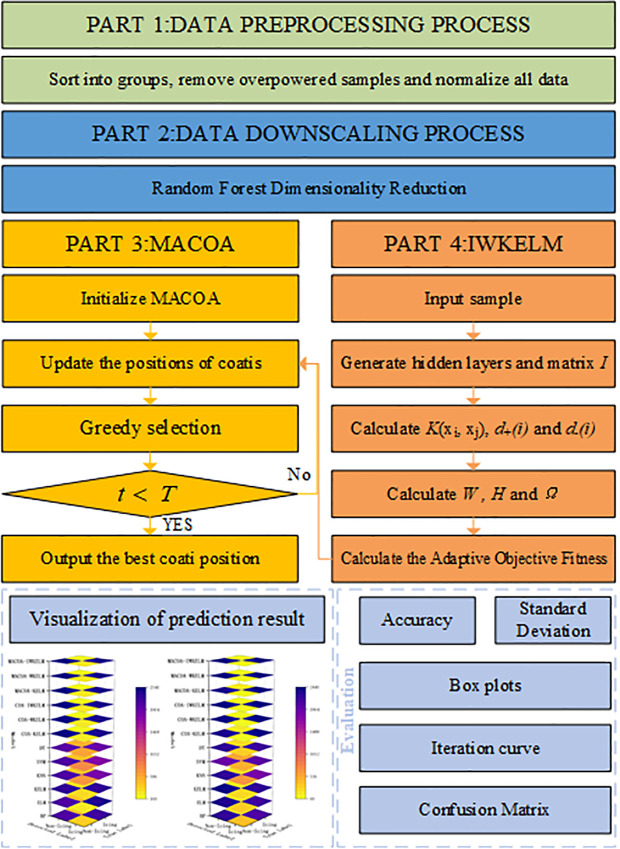
Framework of MACOA-IWKELM.

The framework of MACOA-IWKELM is shown in Fig 5.

## Model diagnostic experiments

### Introduction to the datasets and models

All the experimental conditions are performed in a test environment with AMD R7 CPU, 3.20GHz, 16GB RAM, and Windows 11 64-bit. PCA method is performed using SPSSPRO software. BP neural network, Support Vector Machine (SVM), and Decision Tree (DT) model training are performed using MATLAB toolkit. The k-nearest neighbour (KNN), ELM and their derived models are programmed using MATLAB 2018a.

12 datasets are used in the experiment, which includes datasets 1–4 from UCI and datasets 5–12 from KEEL. All datasets are normalized. The experimental dataset is shown in Table 9: it contains the sample name, source, number of sample features, total number of samples, and number of positive and negative class samples.

**Table 9:** The source and details of the datasets.

No.	Name	Source	Feature Count	Positive Sample Count	Negative Sample Count
1	blood_transfusion	UCI	4	570	178
2	banknote_authentication	UCI	4	762	610
3	Statlog (Heart)	UCI	13	150	120
4	Vertebral_Column	UCI	6	210	100
5	Pima	KEEL	8	500	268
6	ionosphere	KEEL	33	225	126
7	magic	KEEL	10	12322	6688
8	phoneme	KEEL	5	3818	1586
9	ring	KEEL	20	3736	3664
10	spambase	KEEL	57	2785	1812
11	twonorm	KEEL	20	3703	3697
12	wdbc	KEEL	30	357	212

A total of 12 models, BP, ELM, KELM, KNN, SVM, DT, COA-KELM, MACOA-KELM, COA-WKELM, MACOA-WKELM, COA-IWKELM, MACOA-IWKELM are used for the comparison experiments in this experiment. Where COA-KELM is the KELM optimized by COA, MACOA-WKELM is the WKELM optimized by MACOA, and so on.

100 training samples and 100 test samples are randomly selected in the data set for each experiment, with half of the samples in each of the positive and negative categories. In each experiment, all models use this randomly selected data at the same time. A total of 50 experiments are conducted, and the experimental results are averaged.

In this experiment, because the data used is test data set, the test function in MACOA experiment has higher complexity, so the maximum number of iterations need not be set too high. The experimental hardware conditions are shown in Table 3. The population size and maximum number of iterations are set to 20 and 200. The model fixed parameters and particle optimization ranges are shown in Table 10.

**Table 10:** Values set for control parameters of compared model.

Model	Parameter	Value
BP	*epochs*	1000
	*goal*	0.0001
	*η*	0.001
	*LL*:Number of hidden layers	5
ELM	*LL*:Number of hidden layers	100
	*C*	100
KELM	*LL*:Number of hidden layers	100
	*g*	1
KNN	*k*	3
SVM	Kernel function	gaussian
	Box Constraint	1
	Kernel Scale	1
DT	Max NumSplits	Inf
	Min Leaf Size	1
	Max Depth	Inf
	Splitcriterion	gdi
COA-KELM COA-WKELM COA-IWKELM MACOA-KELM MACOA-WKELM MACOA-IWKELM	*r*:random number	[0,1]
	*I*:random number	{0,1}

### Results of diagnostic experiments on the dataset

**Fig 6:**
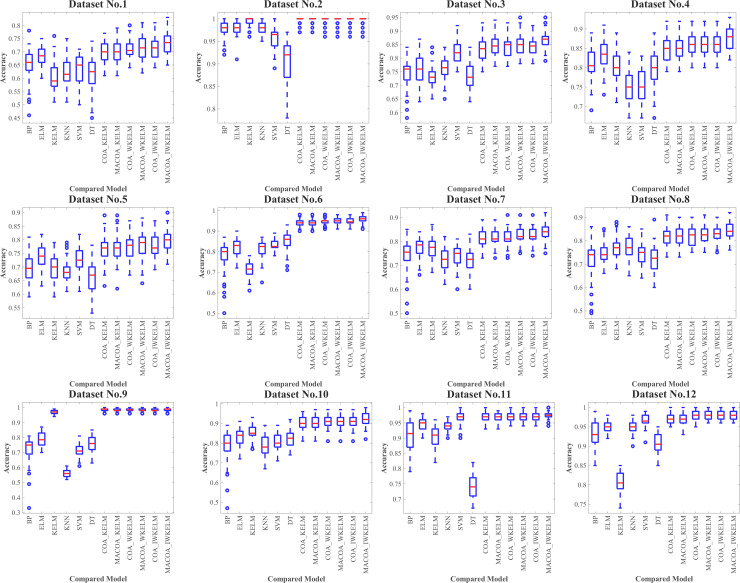
Box plot of the compared models for Dataset1-12 in diagnostic experiment.

In order to confirm that MACOA and IWKELM can improve the classification effect when optimizing the model parameters, datasets 1–12 are selected for the experiment. The experimental results are presented in Tables 11 and 12. Among them, the distribution of 50 experiments is shown in the box plot Fig 6.

**Table 11:** Accuracy of the compared models for Dataset1-12 in diagnostic experiment.

Name	No.												Average
	1	2	3	4	5	6	7	8	9	10	11	12	
BP	65.00%	98.17%	73.93%	81.43%	67.23%	78.83%	70.10%	71.37%	70.63%	79.53%	89.77%	91.97%	78.16%
ELM	67.27%	98.10%	77.10%	83.50%	71.43%	82.60%	76.40%	74.33%	77.87%	83.87%	95.37%	94.50%	81.86%
KELM	60.57%	98.77%	72.40%	80.17%	68.50%	72.30%	76.47%	77.37%	96.40%	86.47%	91.07%	79.07%	79.96%
KNN	63.67%	98.40%	76.70%	73.83%	67.23%	80.90%	72.17%	76.87%	55.60%	78.60%	94.63%	94.83%	77.79%
SVM	62.93%	96.37%	80.87%	75.73%	71.97%	83.80%	73.87%	74.33%	71.07%	81.70%	97.07%	95.63%	80.44%
DT	61.83%	91.57%	72.20%	79.23%	67.07%	85.63%	70.63%	72.87%	73.33%	80.80%	73.33%	89.90%	76.53%
COA-KELM	69.57%	99.43%	82.70%	84.77%	74.87%	94.07%	81.07%	81.83%	97.87%	90.70%	97.13%	96.43%	87.54%
MACOA-KELM	69.73%	99.40%	83.67%	85.10%	75.47%	93.90%	81.37%	81.97%	97.87%	90.63%	97.07%	96.40%	87.71%
COA-WKELM	70.83%	99.77%	83.13%	85.83%	76.10%	94.57%	81.50%	82.50%	98.13%	91.20%	97.57%	97.00%	88.18%
MACOA-WKELM	71.43%	99.80%	84.57%	**86.20%**	76.77%	**94.60%**	82.40%	82.67%	98.03%	91.13%	97.70%	97.07%	88.53%
COA-IWKELM	71.10%	**99.83%**	84.10%	85.73%	76.33%	94.53%	82.03%	82.90%	98.10%	91.20%	97.73%	97.17%	88.40%
MACOA-IWKELM	**72.73%**	99.80%	**84.80%**	86.17%	**77.33%**	94.50%	**82.87%**	**83.57%**	**98.27%**	**91.40%**	**97.80%**	**97.33%**	**88.88%**

**Table 12:** Standard deviation of the compared models for Dataset1-12 in diagnostic experiment.

Name	No.												
1	2	3	4	5	6	7	8	9	10	11	12	Average
BP	5.57%	1.62%	8.01%	4.34%	7.84%	7.23%	8.81%	8.24%	6.02%	6.26%	4.30%	5.03%	6.11%
ELM	3.49%	1.75%	3.42%	3.65%	5.69%	4.68%	3.54%	3.77%	3.82%	4.83%	2.11%	2.01%	3.56%
KELM	5.67%	1.19%	3.16%	3.38%	6.06%	3.31%	4.55%	3.93%	1.99%	3.43%	1.95%	3.29%	3.49%
KNN	5.00%	1.45%	3.97%	2.97%	5.69%	5.14%	4.82%	4.80%	2.47%	3.97%	2.11%	1.78%	3.68%
SVM	5.35%	1.81%	3.23%	4.12%	5.18%	4.45%	4.45%	3.74%	4.09%	4.32%	1.28%	1.81%	3.65%
DT	5.61%	3.45%	3.93%	3.87%	5.36%	4.15%	4.88%	3.79%	4.50%	4.34%	4.40%	3.14%	4.29%
COA-KELM	3.87%	0.82%	3.34%	3.17%	4.94%	2.46%	3.27%	3.59%	1.41%	3.58%	1.59%	1.74%	2.82%
MACOA-KELM	3.69%	0.81%	3.39%	2.93%	4.21%	2.47%	3.23%	3.45%	1.41%	3.34%	1.46%	1.81%	2.68%
COA-WKELM	3.80%	0.50%	3.22%	2.74%	**3.21%**	2.42%	3.27%	3.16%	1.36%	3.42%	1.43%	1.78%	2.53%
MACOA-WKELM	3.55%	0.48%	3.16%	2.48%	4.11%	2.40%	3.18%	3.31%	1.25%	3.54%	1.60%	1.72%	2.57%
COA-IWKELM	3.88%	0.46%	3.02%	2.78%	3.76%	2.45%	3.27%	3.28%	1.24%	3.64%	1.34%	1.64%	2.56%
MACOA-IWKELM	**3.30%**	**0.28%**	**2.88%**	**2.44%**	4.09%	**2.32%**	**2.50%**	**3.04%**	**1.03%**	**3.27%**	**1.22%**	**1.43%**	**2.32%**

In this experiment, since there are more models and more combinations, a side-by-side comparison is needed, so some of the models are combined to facilitate the comparison, and the groups set are as follows:

Group 1: BP, ELM, KELM, KNN, SVM, DT, COA-KELM
Group 2: COA-KELM, MACOA-KELM
Group 3: COA-WKELM, MACOA-WKELM
Group 4: COA-IWKELM, MACOA-IWKELM
Group 5: COA-KELM, COA-WKELM, COA-IWKELM
Group 6: MACOA-KELM, MACOA-WKELM, MACOA-IWKELM

It can be concluded from the results of classification correctness in Table 11, and classification accuracy variance in Table 12, and box plot in the Fig 6.

Group 1 is selected for comparison, and the results indicate that the BP, ELM, KELM, KNN, SVM, and DT models do not achieve a high classification accuracy. The highest average accuracy reaches only 81.86%, with the lowest average standard deviation at just 3.49%. In contrast, the average accuracy of the COA-KELM model is 87.54%, significantly higher than the KELM model’s accuracy of 79.96%. This discrepancy arises because the traditional model lacks optimization of its parameters, which hinders improvements in classification performance and reduces stability. Additionally, as shown in the box plot in Fig 6, the traditional model exhibits more outliers and larger classification errors.

The comparisons in groups 2, 3, and 4 reveal that the average accuracy of MACOA-KELM reaches 87.71%, which is 0.17% higher than the average accuracy of COA-KELM. Additionally, the average standard deviation is only 2.68%, which is 0.14% lower than that of the COA-KELM model. Furthermore, the average accuracy of MACOA-WKELM is 88.53%, representing a 0.35% improvement over the average accuracy of COA-WKELM, with an average standard deviation of just 2.57%. The MACOA-IWKELM model achieves an average accuracy of 88.88%, which is 0.48% higher than the average accuracy of COA-IWKELM, and an average standard deviation of only 2.32%, which is 0.24% lower than that of the COA-KELM model.

Overall, the MACOA demonstrates a higher correct classification rate and a smaller standard deviation compared to the COA, effectively improving stability. This improvement is attributed to the initial population generated by the Lévy flight, which is more conducive to optimization, and the optimization speed is significantly enhanced by the nonlinear factor. Additionally, the proposed coati vigilance mechanism ensures that the algorithm can escape local optima and avoid missing the global optimum. Furthermore, the optimized objective function enhances the optimization logic and provides a solution when the original iteration fails to yield a better value. The box plot also illustrates that MACOA exhibits significant superiority and stability.

From the comparative models in groups 5 and 6, COA-WKELM achieves an average accuracy of 88.18%, which is 0.64% higher than the average accuracy of COA-KELM. The average standard deviation is only 2.53%, which is 0.29% lower than that of COA-KELM. COA-IWKELM achieves an average accuracy of 88.40%, which is 0.22% higher than the average accuracy of COA-WKELM. The average accuracy of MACOA-WKELM reaches 88.53%, representing an increase of 0.82% over the accuracy of MACOA-KELM, while the average standard deviation of MACOA-WKELM is only 2.57%, which is 0.11% lower than that of MACOA-KELM. Furthermore, the average accuracy of MACOA-IWKELM reaches 88.88%, which is 0.35% higher than that of MACOA-WKELM, and the average standard deviation of MACOA-IWKELM is only 2.32%, which is 0.25% lower than that of MACOA-WKELM.

Therefore, the weight parameters introduced into the IWKELM can further enhance classification accuracy. Additionally, the box plot demonstrates that IWKELM significantly increases the stability of multiple predictions, with very few outliers. However, in some models, the average standard deviation of WKELM was nearly equal to that of IWKELM. This similarity can be attributed to the limitations of certain datasets and the instability caused by the chaotic mapping mechanism. These issues could be mitigated by utilizing more datasets, increasing the number of iterations, and conducting extensive experimentation. Overall, MACOA-IWKELM exhibits superior optimization search speed and convergence compared to the other models.

#### Wind turbine blade icing diagnostic experiment.

The experimental data presented in this paper is sourced from the Industrial Big Data Innovation Competition. The dataset records operational data from November 1, 2015, to January 1, 2016, for two turbines, identified as Turbine 15 and Turbine 21, each containing 20 features.

Before conducting the experiments, the wind turbine operation data were processed to remove duplicates, average the samples with the same timestamp, and eliminate samples with power outputs greater than 2 kW. This resulted in 39,465 normal samples and 2,841 icing samples for Turbine 15, and 17,602 normal samples and 1,274 icing samples for Turbine 21. Subsequently, the blade pitch angle, blade pitch speed, and pitch motor temperature data were averaged to yield a total of 20 features. The dataset information is summarized in Table 13, while the corresponding attribute numbers for the wind turbine blade operation data are detailed in Table 14.

**Table 13:** The source of the fan datasets and details.

No.	Name	Source	Feature Count	Sample Count	Positive Sample Count	Negative Sample Count
1	15wind	The First Industrial Big Data Innovation Competition	20	13607	10766	2841
2	21wind		20	5058	3784	1274

**Table 14:** Number of corresponding attributes of fan operation data.

Feature No.	1	2	3	4	5
Feature name	Wind Speed	Generator RPM	Output Power	Wind Direction	Wind Direction (25s)
Feature No.	6	7	8	9	10
Feature name	Yaw Position	Yaw Rate	Average Pitch Angle	Average Pitch Rate	Average Pitch Motor emperature
Feature No.	11	12	13	14	15
Feature name	Acceleration in X Direction	Acceleration in Y Direction	Ambient Temperature	Cabin Temperature	1_ng5_tmp
Feature No.	16	17	18	19	20
Feature name	2_ng5_tmp	3_ng5_tmp	1_ng5_DC	2_ng5_DC	3_ng5_DC

#### Random forest dimensionality reduction.

Random Forest (RF) Dimensionality Reduction is a feature selection and dimensionality reduction technique based on the Random Forest algorithm [40]. In terms of dimensionality reduction, Random Forest effectively identifies and selects the features that have the greatest impact on the target variable, thereby reducing the dimensionality of the data.

**Fig 7:**
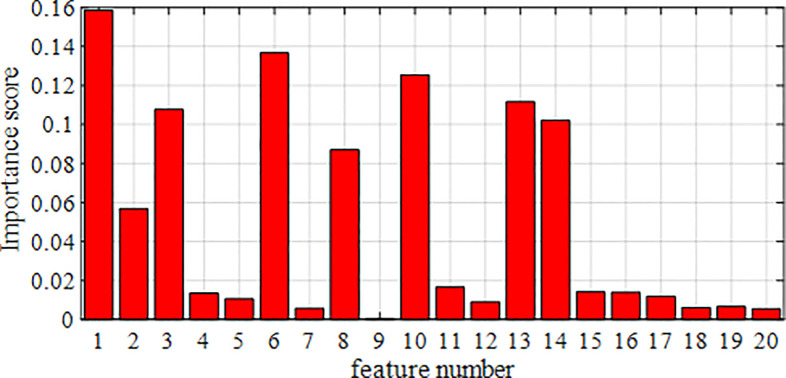
Importance of the attributes of fan No.15.

**Fig 8:**
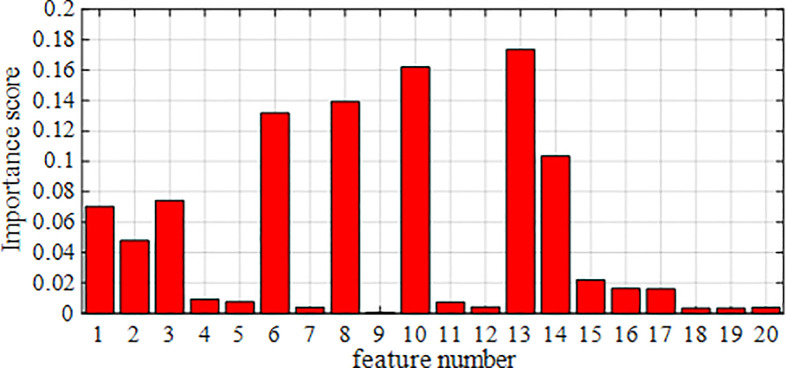
Importance of the attributes of fan No.21.

**Fig 9:**
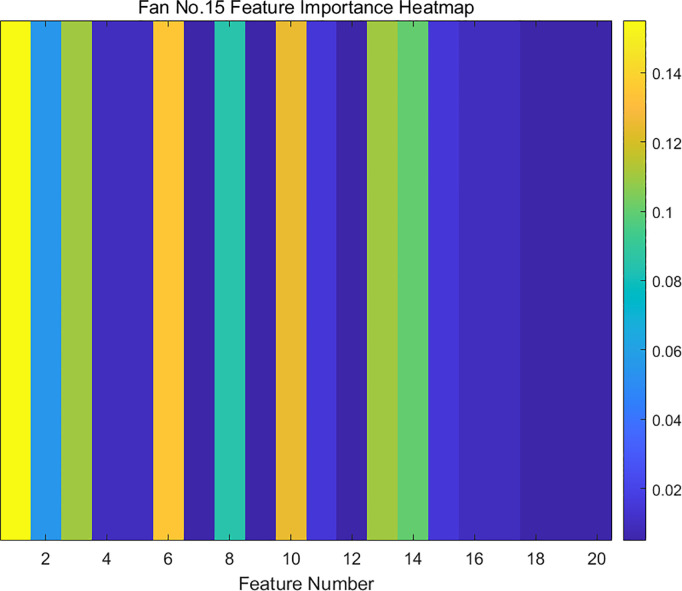
Feature importance heat map of fan No.15.

**Fig 10:**
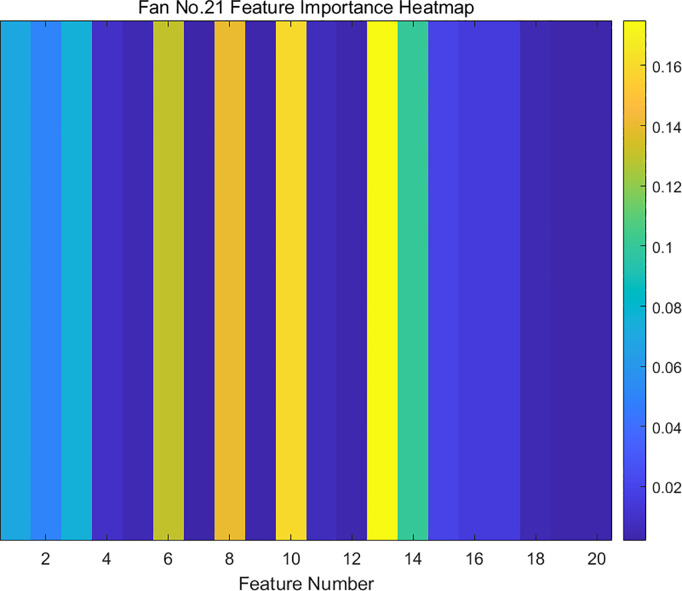
Feature importance heat map of fan No.21.

The SCADA data of wind turbine blades are processed by RF dimensionality reduction. The specifics of the attribute scores of the SCADA data for turbine 15 and 21 operation under the use of the RF method are shown in Figs 7 and 8. The feature importance heat map drawn based on feature importance is shown in Figs 9 and 10.

Based on the results presented in Figs 7–10, the importance of the top 8 attributes for Turbines 15 and 21 is significantly greater than that of the other attributes. In particular, the importance of the eighth-ranked feature, Generator RPM, is three times that of the ninth-ranked feature. Therefore, experiments were conducted on datasets with 8 or fewer extracted features.

Therefore, based on the experimental results in Tables 15 and 16, this paper selects the top 8 features with the highest scores as the input feature vectors for each experimental model, while the other attributes are disregarded. The top 8 highest-scoring features are wind speed, yaw position, average pitch motor temperature, ambient temperature, output power, cabin temperature, average pitch angle, and generator RPM.

**Table 15:** The impact of the number of selected features in diagnostic experiments for Fan No.15.

Model	Number of features							
	1	2	3	4	5	6	7	8
BP	50.33%	52.42%	55.83%	58.17%	55.83%	54.50%	55.00%	81.76%
ELM	51.00%	53.92%	59.67%	64.50%	64.83%	59.83%	63.33%	84.72%
KELM	52.42%	58.92%	60.50%	66.25%	64.33%	61.25%	63.92%	87.44%
KNN	50.92%	55.00%	54.50%	55.58%	57.25%	53.42%	61.33%	76.96%
SVM	50.67%	53.58%	52.42%	54.42%	54.33%	52.00%	53.58%	75.06%
DT	51.83%	56.33%	56.92%	62.67%	56.42%	59.17%	58.08%	76.66%
COA_KELM	59.33%	65.00%	66.67%	70.42%	69.92%	67.75%	70.00%	90.32%
MACOA_KELM	60.00%	65.25%	66.50%	70.42%	69.92%	67.50%	70.17%	90.20%
COA_WKELM	59.92%	66.00%	67.00%	71.42%	71.00%	68.58%	70.42%	91.00%
MACOA_WKELM	59.50%	66.08%	66.92%	71.58%	70.75%	69.08%	70.83%	90.84%
COA_IWKELM	61.00%	66.58%	67.25%	71.67%	70.92%	69.08%	70.42%	91.10%
MACOA-IWKELM	60.75%	66.33%	67.75%	72.25%	71.42%	69.25%	71.08%	91.22%

**Table 16:** The impact of the number of selected features in diagnostic experiments for Fan No.21.

Model	Number of features							
	1	2	3	4	5	6	7	8
BP	74.77%	76.77%	81.15%	81.31%	78.69%	79.46%	76.38%	85.84%
ELM	76.38%	77.00%	80.23%	79.69%	79.23%	81.46%	79.23%	89.54%
KELM	70.31%	80.00%	85.77%	87.31%	89.62%	86.54%	85.15%	92.82%
KNN	70.62%	78.15%	80.15%	80.77%	81.46%	81.08%	79.62%	81.76%
SVM	76.38%	77.54%	78.31%	78.92%	76.69%	78.23%	77.77%	78.28%
DT	69.15%	79.23%	83.08%	83.38%	82.15%	83.69%	81.38%	87.42%
COA_KELM	77.38%	85.38%	88.85%	90.00%	91.69%	89.46%	88.54%	95.52%
MACOA_KELM	77.31%	85.38%	88.77%	89.85%	91.69%	89.38%	88.46%	95.48%
COA_WKELM	77.31%	86.31%	89.15%	90.77%	92.15%	90.38%	89.08%	96.00%
MACOA_WKELM	78.00%	86.23%	89.54%	90.69%	92.46%	90.31%	88.85%	95.86%
COA_IWKELM	77.31%	87.08%	89.38%	90.85%	92.54%	90.31%	89.46%	96.06%
MACOA-IWKELM	77.77%	86.77%	89.77%	90.85%	92.54%	90.31%	89.15%	96.94%

#### Diagnostic results and comparative analysis of MACOA-IWKELM.

The SCADA data from two turbines were downscaled and then processed using the SMOTE oversampling technique, resulting in 39,465 normal samples and 2,841 icing samples for Turbine 15, and 17,602 normal samples and 1,274 icing samples for Turbine 21.

The processed data is then fed into the classification models for experimentation. The experimental comparison models include BP, ELM, KELM, SVM, KNN, COA-KELM, MACOA-KELM, COA-WKELM, MACOA-WKELM, COA-IWKELM, and MACOA-IWKELM, totaling 12 models.

The fixed parameters for the experimental models and the optimization algorithm’s search range are consistent with those in Section 6.1. The experimental hardware conditions are shown in Table 3. The population size and maximum number of iterations are set to 20 and 200. The model fixed parameters and particle optimization ranges are shown in Table 10.

**Fig 11:**
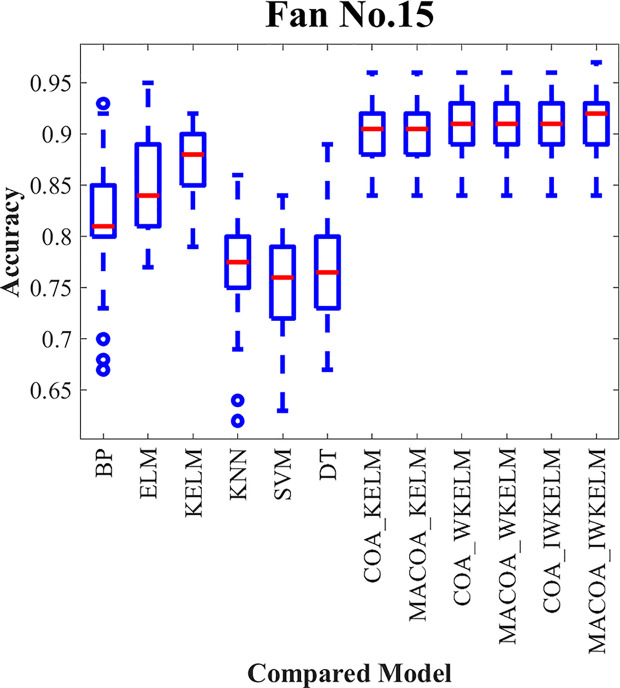
Box plot of the compared models for Fan No.15 in diagnostic experiment.

**Fig 12:**
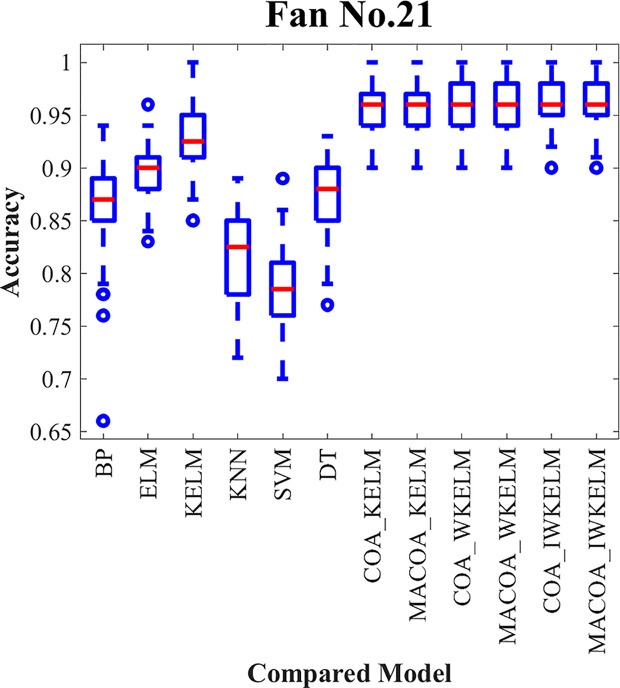
Box plot of the compared models for Fan No.21 in diagnostic experiment.

**Fig 13:**
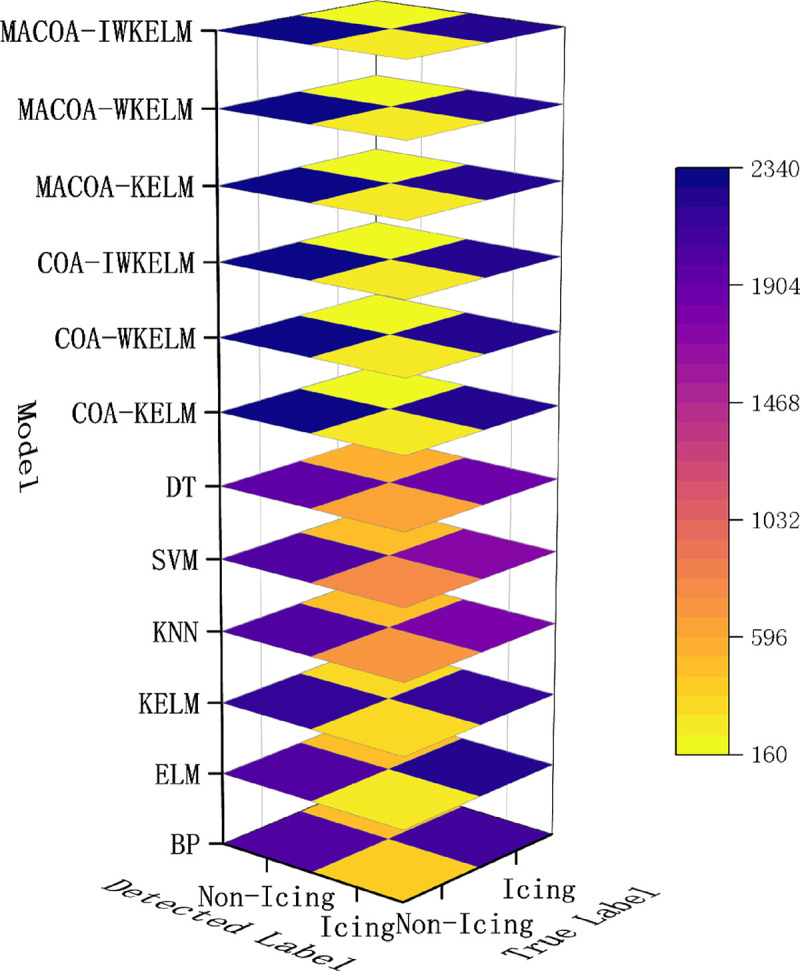
Confusion Matrix of the compared models for Fan No.15 in diagnostic experiment.

**Fig 14:**
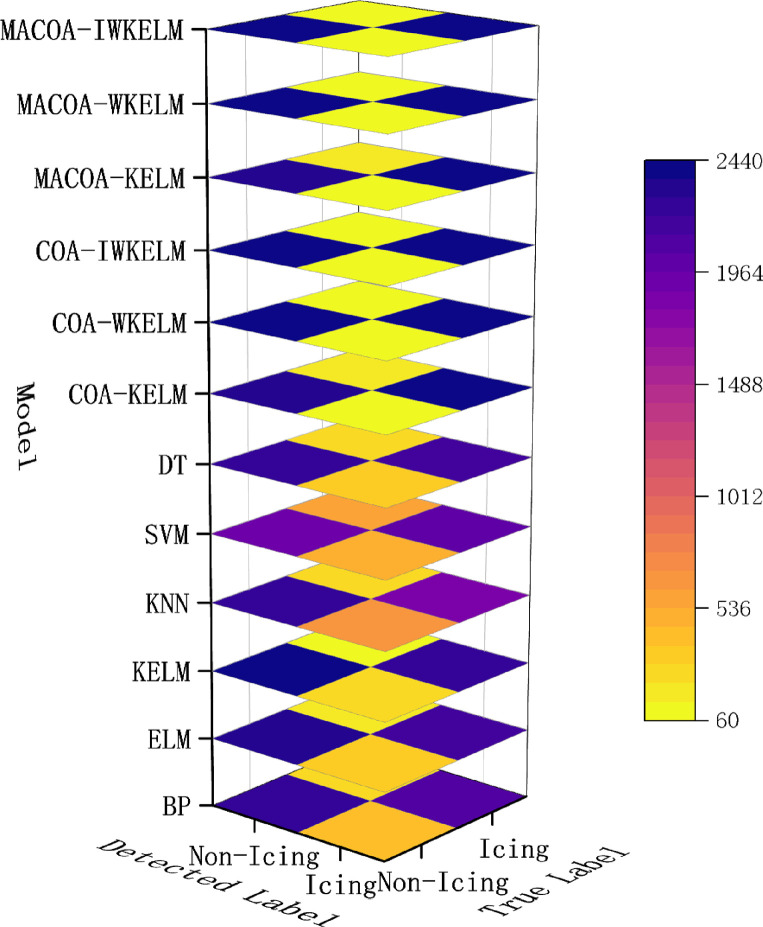
Confusion Matrix of the compared models for Fan No.21 in diagnostic experiment.

The diagnostic accuracy of the experiment for wind turbine No.15 and No.21 is shown in Tables 17–19, where the distribution of the 50 experiments is shown in the box plot Figs 11 and 12, and the confusion matrices generated by the diagnostic experiments for wind turbine 15 and wind turbine 21 out of the 50 experiments are shown in Figs 13 and 14.

**Table 17:** Results of the compared models for Fan No.15 and Fan No.21 in diagnostic experiment.

Model	Fan No.15				Fan No.21			
	TP	TN	FP	FN	TP	TN	FP	FN
BP	2037	2051	463	449	2208	2084	292	416
ELM	2039	2197	461	303	2297	2180	203	320
KELM	2187	2185	313	315	2367	2274	133	226
KNN	2047	1801	453	699	2220	1868	280	632
SVM	2017	1736	483	764	1938	1976	562	524
DT	1946	1887	554	613	2209	2162	291	338
COA_KELM	2286	2230	270	214	2349	2427	73	151
MACOA_KELM	2288	2222	278	212	2357	2417	83	143
COA_WKELM	2331	2219	281	169	2371	2429	71	129
MACOA_WKELM	2321	2221	279	179	2367	2426	74	133
COA_IWKELM	2325	2230	270	175	2376	2427	73	124
MACOA-IWKELM	2327	2234	266	173	2411	2436	44	109

**Table 18:** Evaluation of the compared models for Fan No.15 and Fan No.21 in diagnostic experiment.

Model	Fan No.15			Fan No.21		
	precision	recall	F1-score	precision	recall	F1-score
BP	81.48%	81.94%	81.71%	88.32%	84.15%	86.18%
ELM	81.56%	87.06%	84.22%	91.88%	87.77%	89.78%
KELM	87.48%	87.41%	87.45%	94.68%	91.28%	92.95%
KNN	81.88%	74.54%	78.04%	88.80%	77.84%	82.96%
SVM	80.68%	72.53%	76.39%	77.52%	78.72%	78.11%
DT	77.84%	76.05%	76.93%	88.36%	86.73%	87.54%
COA_KELM	89.44%	91.44%	90.43%	96.99%	93.96%	95.45%
MACOA_KELM	89.17%	91.52%	90.33%	96.60%	94.28%	95.43%
COA_WKELM	89.24%	93.24%	91.20%	97.09%	94.84%	95.95%
MACOA_WKELM	89.27%	92.84%	91.02%	96.97%	94.68%	95.81%
COA_IWKELM	89.60%	93.00%	91.27%	97.02%	95.04%	96.02%
MACOA-IWKELM	89.74%	93.08%	91.38%	98.21%	95.67%	96.92%

**Table 19:** Accuracy and standard deviation of the compared models for Fan No.15 and No.21.

Model	Fan No.15		Fan No.21	
	Accuracy	Standard Deviation	Accuracy	Standard Deviation
BP	81.76%	5.52%	85.84%	4.72%
ELM	84.72%	4.53%	89.54%	2.87%
KELM	87.44%	3.23%	92.82%	3.24%
KNN	76.96%	4.85%	81.76%	4.07%
SVM	75.06%	5.12%	78.28%	3.96%
DT	76.66%	5.10%	87.42%	3.81%
COA_KELM	90.32%	2.94%	95.52%	2.31%
MACOA_KELM	90.20%	2.86%	95.48%	2.33%
COA_WKELM	91.00%	2.87%	96.00%	2.24%
MACOA_WKELM	90.84%	2.87%	95.86%	2.26%
COA_IWKELM	91.10%	2.81%	96.06%	2.23%
MACOA-IWKELM	**91.22%**	**2.53%**	**96.94%**	**1.92%**

According to the evaluation indicators in Table 18, in the experiment of fan No.15, the indicators of the COA_KELM model exceeded those of all traditional models. Meanwhile, the F1 score of MACOA_WKELM is 1.32% higher than that of MACOA_KELM, while the F1 score of MACOA_IWKELM is 0.36% higher than that of MACOA_WKELM. In addition, in the experiment of fan No. 21, all indicators of COA_KELM were superior to those of the traditional model. The F1 score of COA_WKELM was 0.50% higher than that of COA_KELM. The F1 score of COA_IWKELM was 0.07% higher than that of COA_WKELM. This proves the effectiveness of IWKELM’s improvements. In addition, in both experiments, the F1 score of MACOA_IWKELM was 0.11% and 0.90% higher than that of COA_IWKELM, respectively. This proves the superiority of MACOA over COA.

From the results presented in Tables 17 and 19 and the box plot of the distribution of 50 experiments shown in Fig 11 and 12. The prediction accuracy of MACOA-KELM for Turbine No. 15 and Turbine No. 21 reach 90.20% and 95.48%, respectively, both of which are significantly higher than those of traditional models such as BP and ELM. Moreover, the standard deviations of the 50 predictions for Turbines No. 15 and No. 21 are only 2.86% and 2.33%, respectively, which are much smaller than those of the traditional models. The accuracy of MACOA-IWKELM is 0.12% and 0.88% higher than that of COA-IWKELM for Turbines 15 and 21, respectively. Additionally, the standard deviations of the 50 predictions for MACOA-IWKELM are only 2.53% and 1.92%, which are lower than the standard deviations of the 50 experiments for COA-IWKELM on Turbines 15 and 21 by 0.28% and 0.31%, respectively. Therefore, it can be concluded that MACOA significantly improves prediction accuracy by applying the chaotic mapping mechanism, nonlinear inertia weighting factors, an improved sparrow vigilante mechanism, and an enhanced objective function. Regardless of whether the optimized model is KELM, WKELM, or IWKELM, both the correct classification rate and the stability of the experimental data are significantly improved compared to using the original COA.

The experimental results indicate that in the Fan No. 15 experiment, the prediction accuracy of MACOA-IWKELM is 0.38% higher than that of MACOA-WKELM, while the standard deviation is 0.28% lower. Additionally, in the Fan No. 21 experiment, the prediction accuracy of MACOA-IWKELM is 0.88% higher than that of MACOA-WKELM, with a standard deviation that is 0.31% lower. Therefore, IWKELM can significantly enhance prediction accuracy when handling data with more features, thanks to the inclusion of a weight parameter that varies according to the individual samples. In conclusion, both MACOA and IWKELM improve the accuracy and stability of fault diagnosis for wind turbine blade icing.

## Conclusion and future prospects

To improve diagnostic accuracy, a wind turbine blade icing fault diagnosis model based on MACOA-IWKELM is proposed. Firstly, weight parameters are introduced into the method, allowing them to be adjusted according to the internal distribution of samples, thereby leading to the development of the IWKELM model. Additionally, to enhance the convergence performance and stability of the Coati Optimization Algorithm (COA), chaotic mapping Lévy flight is employed to optimize the initial population, and nonlinear inertia weight factors are added to improve convergence speed. The vigilante mechanism of the improved sparrow optimization algorithm is utilized to enhance stability. The performance of the Coati Optimization Algorithm is significantly improved by incorporating the enhanced objective function during the iteration process.

The effectiveness of MACOA is validated through comparative experiments, which demonstrate that the multi-strategy adaptive Coati Optimization Algorithm outperforms the other 11 comparison algorithms. MACOA is used to optimize IWKELM, resulting in the proposed MACOA-IWKELM model. Experiments conducted with 12 publicly available datasets from UCI and KEEL indicate that the model significantly enhances classification accuracy and stability. Finally, the MACOA-IWKELM model is applied to diagnose faults in two sets of real turbine operation data. Based on the experimental results, the improved model shows a significant increase in fault diagnosis accuracy and stability.

However, the proposed model does have some limitations, primarily related to the parameter settings for population size and maximum number of iterations, which are based on empirical values. In the future, further optimization of the model will be necessary to achieve even better diagnostic results.
